# Glances at the Hospitals

**Published:** 1899-12-02

**Authors:** 


### GLANCES AT THE HOSPITALS.

### THE CITY HOSPITAL, BOSTON, MASS.

This is a large hospital, and can accommodate at least 800 patients. It is constructed on the block principle. The hospital contained 488 patients at the beginning of the year, and there were admitted during the twelve months 3,926 medical cases, 3,494 surgical, 558 gynæcological, 107 ophthalmic, and 146 aural cases. The average number of days' stay of patients was only 20, and the entire amount expended for all departments of the hospital during the year was 387,527 dollars. A training school for nurses is attached to the hospital, and is now in its twenty-first year of existence. Altogether 535 nurses have obtained certificates. At first the course was of two years' duration only, but now a third year has been added, and many of the two-years' certificated nurses are voluntarily submitting to the third year of study. It is interesting to note the various nationalities admitted to United States hospitals. What a curious mixture the clientèle is! In addition to the natives of the United States and British American provinces, there were in the year's admissions upwards of 1,000 Irishmen, 300 English, 113 Scotch, 271 Italians, 233 Russians, 158 Germans, 101 Swedes, and smaller numbers of Poles, Norwegians, Danes, Fins, Dutch, Turks, Chinese, Portuguese, Greeks, &c. The hospital is extremely well manned from a medical point of view. In addition to the house physicians and house surgeons there are sixty-nine medical men on the staff. The report concludes with a list of the nurses who have obtained certificates since these were first given. This is a point that might be advantageously copied by our English hospitals.

